# The relationship between sleep quality and occupational identity among psychiatric night shift nurses

**DOI:** 10.3389/fpsyt.2026.1711613

**Published:** 2026-02-26

**Authors:** Qianqian Qin, Haiyan Zhu, Mu Yu, Juan Bao, Zejun Ma, Xueyan Zhu, Kaihong Tang, Peiyun Zhang

**Affiliations:** Department of Psychiatry, Nantong Fourth People’s Hospital, Nantong, Jiangsu, China

**Keywords:** influencing factors, night shift nurses, occupational identity, psychiatric nursing, sleep quality

## Abstract

**Purpose:**

This study aimed to investigate the relationship between sleep quality and occupational identity among psychiatric night shift nurses.

**Methods:**

This cross-sectional survey was conducted from August to December 2024 among psychiatric night shift nurses at Nantong Fourth People’s Hospital. Data were collected using a self-administered questionnaire covering three domains: (1) sociodemographics and work characteristics, (2) lifestyle factors (physical exercise and dietary regularity), and (3) night shift related sleep patterns. The survey used the Pittsburgh Sleep Quality Index and the Occupational Identity Scale. Analysis of variance and logistic regression were used to identify factors influencing sleep quality. Pearson’s correlation analysis was used to examine the relationship between sleep quality and occupational identity.

**Results:**

Logistic regression analysis indicated that the following factors were significantly associated with sleep quality: night shift frequency, sleep quality before and after shifts, daily diet, daily exercise, and the total score of occupational identity. Subjective sleep quality, sleep latency, sleep duration, sleep disturbance, and daytime dysfunction scores were significantly negatively correlated with total occupational identity score and scores of its dimensions.

**Conclusion:**

Psychiatric night-shift nurses generally exhibited poor sleep quality, and the total PSQI score showed a significant negative correlation with the total OIS score. Key factors influencing sleep quality include night shift frequency, sleep quality before and after shifts, as well as lifestyle factors such as daily diet and regular exercise, and occupational identity. Therefore, nursing managers should prioritize sleep health management by optimizing schedules, promoting healthy lifestyle habits, and enhancing occupational identity to improve job stability.

## Introduction

Global studies have shown that nurses generally experience poor sleep quality, which is closely related to the unique demands of nursing work and its high-pressure environment (1). A recent meta-analysis of observational studies reported that the pooled prevalence of poor sleep quality among nursing staff worldwide is approximately 61.0% (2). A survey of 3,206 nurses in China revealed that 55.1% of those working the night shift had poor sleep quality, underscoring the substantial effect of night shift work patterns on the sleep health of nurses (3). Not only do night shifts disrupt normal circadian rhythms, they also increase the risk of chronic sleep deprivation and sleep disorders (4, 5). Psychiatric nurses experience more severe sleep issues due to their higher shift frequency and stressful work environment (6). Data shows that 71.5% of Chinese psychiatric nurses experience sleep quality issues, which is significantly higher than the rate in the general nursing population (7). Their sleep quality is influenced by multiple factors, including shift frequency, psychological distress, and work-related burnout (6, 8). Of particular concern, nurses in psychiatric closed wards face dual threats to occupational safety and mental health due to frequent exposure to patient aggression (9). A study indicates that for nurses, the experience of workplace violence further exacerbates the risk of sleep disorders (10).

Occupational identity, a central concept in understanding professional engagement, refers to the dynamic integration of cognitive understanding, affective evaluation, and behavioral commitment that an individual holds toward their nursing role and its place within their self-concept (11, 12). For nurses, the development and strength of this identity are shaped by a confluence of factors. It is enhanced by experiences that foster a sense of competence and value, such as professional autonomy, supportive mentorship and peer relationships, the meaningfulness of patient care work, and alignment with organizational values (12, 13). Conversely, it can be weakened or eroded by chronic workplace adversities common in demanding settings like psychiatric nursing and shift work, including role ambiguity, excessive workload, emotional exhaustion, and poor work life balance (14). A stronger occupational identity has been consistently linked to positive outcomes, serving as a buffer against job stress and burnout (15), and is associated with higher job satisfaction, retention rates, and quality of care (16, 17). In recent years, the relationship between occupational well-being and sleep has garnered increased attention. A growing body of research indicates that sleep quality is intertwined with professional factors; for instance, poor sleep is correlated with lower job satisfaction and professional fulfillment among nurses, while better sleep supports resilience and engagement (18, 19). Although previous studies have explored various influencers of nurses’ sleep, research specifically targeting psychiatric night-shift nurses a high-risk subgroup remains limited. Therefore, this study aims to investigate the factors influencing sleep quality among psychiatric night-shift nurses and to explore its connection with occupational identity, thereby providing insights for targeted interventions to safeguard their health and sustain the workforce.

## Methods

### Study design and setting

A cross-sectional survey was conducted between August and December 2024 at Nantong Fourth People’s Hospital, a psychiatric hospital in Jiangsu Province, China.

### Participants

Given the single center design, we employed a convenience sampling method. Participants were night-shift nurses from the psychiatric departments of Nantong Fourth People’s Hospital. The inclusion criteria were as follows: (1) engaged in psychiatric nursing work with night shift assignments, (2) at least one year of work experience, and (3) voluntary participation in this study. The exclusion criteria were: (1) a history of severe sleep disorders (e.g., sleep apnea syndrome); (2) a diagnosed mental illness or cognitive impairment; and (3) the occurrence of a recent major life event.

### Sample size

The sample size was estimated using the standard formula for cross-sectional studies designed to estimate a proportion: $n=\frac{Z_{1-\alpha /2}^{2}\times P\left(1-P\right)}{d^{2}}$, where $Z_{1-\alpha /2}$ is the Z-score corresponding to the desired confidence level (1.96 for 95% confidence), *p* is the estimated prevalence of the condition of interest, and d is the margin of error. Based on a prior study reporting that 71.5% of Chinese psychiatric nurses experienced poor sleep quality (7), we set *p* = 0.715. Therefore, a minimum of 313 participants was required. To achieve this sample size, all eligible psychiatric night-shift nurses working in the target departments of Nantong Fourth People’s Hospital during the study period were invited to participate.

### Study tool

1. A self-administered questionnaire was developed to collect information on participants’ characteristics. It collected data on gender, age, educational level, marital status, years of professional experience, monthly income, and night shift details. Specific data collected included: the number of night shifts per month, the average number of night shifts per week, the average length of a night shift, the average interval between night shifts, sleep quality on the day before and after a night shift, and total years of night shift work. Lifestyle data, such as daily exercise and dietary habits, were also collected. Regular exercise: defined as engaging in moderate physical activity for at least 30 minutes per session, three or more times per week; Regular diet: defined as having three meals a day at fixed times without frequent skipping or delayed eating; Sleep quality the day before and after a night shift was assessed and categorized into three levels: 1) Good: Falling asleep within 30 minutes, no or rare awakenings, feeling refreshed and energetic upon waking. 2) Average: Taking 31–60 minutes to fall asleep, occasional awakenings that do not severely disrupt sleep, feeling somewhat rested but not fully energetic upon waking. 3) Poor: Taking more than 60 minutes to fall asleep, frequent awakenings, being awake for long periods during sleep, or feeling unrefreshed and fatigued upon waking.
2. Pittsburgh Sleep Quality Index (PSQI): The PSQI assesses sleep quality over the past month and includes seven factors: subjective quality of sleep, sleep onset latency, length of sleep, efficiency of sleep, sleep disorders, sleep medicine usage, and dysfunction during the day. Each criterion is evaluated on a scale from 0 to 3, yielding a maximum possible score of 21. An elevated score signifies inferior sleep quality. A total score > 7 was used as the cut-off point to indicate ‘poor sleep quality’. The PSQI has demonstrated good reliability and validity in Chinese populations, and its Cronbach’s α was 0.87 (20, 21).
3. Occupational Identity Scale (OIS): Occupational identity was measured using the Occupational Identity Scale (OIS), a 10-item self-report instrument developed by Tyler and McCallum (1998). The scale comprises three dimensions: Occupational Cognition (4 items), reflecting an individual’s understanding and awareness of their professional role; Occupational Evaluation (3 items), pertaining to affective appraisal and value judgment of one’s profession; and Occupational Impact (3 items), indicating the perceived influence of the profession on self-concept and social relationships. All items are rated on a 5-point Likert scale from 1 (Strongly Disagree) to 5 (Strongly Agree), yielding a total score ranging from 10 to 50, with higher scores representing stronger occupational identity. Each subscale score is derived from the sum of its respective items. The OIS has demonstrated good psychometric properties in prior research, and the Cronbach’s α for the total scale was 0.90 (22).

### Data collection

Following the provision of written informed consent, an electronic questionnaire was distributed via the Wenjuanxing (WJX) platform (www.wjx.cn). Participants were given a specified period to complete the survey anonymously. Among the 313 questionnaires distributed, 12 were excluded due to extensive missing data (>20% of items) or patterned responding, resulting in 301 valid questionnaires (96.1% response rate). For the remaining datasets, missing values were minimal (<1% for any single variable) and were handled by pairwise deletion for correlation analyses and listwise deletion for regression analyses. A *post-hoc* power analysis was conducted based on the final sample size of 301 and the observed correlation coefficient between PSQI and OIS scores. With α set at 0.05 (two-tailed), the statistical power of this study exceeded 99%.

### Ethical approval

This study was approved by the Ethics Committee of Nantong Fourth People’s Hospital (Approval No. 2022-k040). Informed consent was obtained from all participants, who were advised of their right to withdraw from the study at any time. All participation in this research was voluntary. The survey was conducted anonymously, and all participants were assured that their responses would remain confidential.

### Statistical analysis

Descriptive statistics were employed for all data. Categorical variables were presented as frequencies and percentages (%), while continuous variables were expressed as the mean and standard deviation (Mean ± SD). Independent samples t-tests and one-way analyses of variance (ANOVAs) were used to compare sleep quality across different sociodemographic, and lifestyle characteristics. The Pearson correlation coefficient was utilized to examine the relationship between sleep quality and occupational identity. Multivariate logistic regression analysis was applied to explore the factors influencing sleep quality. Prior to the regression analysis, multicollinearity among predictor variables was assessed using the Variance Inflation Factor (VIF), with a threshold of VIF< 5 indicating no significant collinearity. Additionally, a *post-hoc* power analysis was conducted to determine the statistical power of the study based on the final sample size and observed effect sizes. All statistical analyses were performed using SPSS version 25.0 (IBM Corp, NY, USA), with a p-value of less than 0.05 considered statistically significant.

## Results

### Participant characteristics

A total of 301 psychiatric night-shift nurses participated in the study. The demographic and work-related characteristics are presented in **Table 1**. Most participants were female (89.70%) and aged between 18 and 40 years (88.37%). The majority were married (71.76%) and held a bachelor’s degree or higher (86.38%). Years of working experience ranged from 5 to 26 years. Regarding work and shift-related features, 70.10% worked 1–5 night shifts per month, and 14.95% worked an average of ≥3 night shifts per week. Additionally, 33.22% worked shifts longer than 8 hours, and 68.77% had more than 5 years of night-shift experience. Concerning lifestyle factors, 85.38% did not engage in regular daily exercise, and 52.49% reported irregular eating habits. Regarding sleep quality, 81.06% of nurses were classified as having poor sleep quality based on a PSQI total score > 7. Total and dimension scores of the OIS and PSQI are presented in **Table 2**.

**Table 1:** Univariate analysis of sleep quality among psychiatric night shift nurses with different demographic characteristics and occupational characteristics (n = 301).

Variable	Group	N(%)	PSQI (Mean ± SD)	t/F	P
Gender	Male	31(10.30%)	10.48 ± 3.19	-0.340	0.734
	Female	270(89.70%)	10.31 ± 2.66		
Age	18-30	125(41.53%)	10.46 ± 2.42	0.716	0.490
	31-40	141(46.84%)	10.34 ± 2.77		
	≥40	35(11.63%)	9.86 ± 2.68		
Education	Vocational school/Junior college	41(13.62%)	10.76 ± 2.17	2.684	0.070
	Undergraduate	259(86.05%)	10.29 ± 2.66		
	Graduate degree or higher	1(0.33%)	5.00 ± 0.00		
Marriage	Single	77(25.58%)	10.79 ± 2.05	1.998	0.137
	Married	216(71.76%)	10.2 ± 2.69		
	Divorced	8(2.66%)	9.38 ± 4.75		
Length of time in work(year)	≤5	61(20.27%)	10.31 ± 2.46	0.726	0.537
	6-10	87(28.90%)	10.66 ± 2.54		
	11-20	128(42.52%)	10.20 ± 2.79		
	21-25	14(4.65%)	10.00 ± 2.04		
	≥26	25(8.31%)	9.96 ± 2.37		
Number of night shifts per month	1-5	211(70.10%)	10.14 ± 2.62	3.900	0.021
	≥6	90(29.90%)	10.79 ± 2.58		
Average number of night shifts per week	1	143(47.51%)	9.85 ± 2.65	5.308	0.005
	2	113(37.54%)	10.65 ± 2.63		
	≥3	45(14.95%)	11.09 ± 2.21		
Average length of night shift (hours)	≤8	201(66.78%)	10.04 ± 2.76	3.900	0.021
	9-16	100(33.22%)	10.89 ± 2.22		
Average number of days between night shifts (days)	≤5	160(53.16%)	10.41 ± 2.67	2.039	0.132
	6-9	115(38.21%)	10.44 ± 2.57		
	≥10	26(8.64%)	9.35 ± 2.40		
Sleep quality the day before a night shift	Good	63(20.93%)	8.89 ± 2.83	18.411	0.000
	Average	141(46.84%)	10.3 ± 2.60		
	Poor	97(32.23%)	11.32 ± 2.00		
Sleep quality the day after a night shift	Good	63(20.93%)	9.46 ± 2.82	9.541	0.000
	Average	144(47.84%)	10.24 ± 2.51		
	Poor	85(28.24%)	11.22 ± 2.34		
Years of night shift work (years)	≤5	94(31.23%)	10.21 ± 2.59	0.699	0.498
	6-9	84(27.91%)	10.62 ± 2.59		
	≥10	123(40.86%)	10.23 ± 2.67		
Daily exercise	No	257(85.38%)	10.55 ± 2.52	3.533	0.000
	Yes	44(14.62%)	9.07 ± 2.86		
Daily diet	Regular	143(47.51%)	9.71 ± 2.69	-4.000	0.000
	Irregular	158(52.49%)	10.89 ± 2.42		
Monthly income (in RMB)	<5000	46(15.28%)	10.63 ± 2.98	2.339	0.098
	5000-8000	215(71.43%)	10.42 ± 2.52		
	>8000	40(13.29%)	9.52 ± 2.61		

PSQI, Pittsburgh Sleep Quality Index; OIS, Occupational Identity Scale; SD, Standard Deviation.

**Table 2:** Total and dimension scores of the OIS and PSQI for psychiatric night shift nurses (n = 301).

Variable	Mean ± SD	Score range
Occupational cognition	10.12 ± 2.65	3-15
Occupational evaluation	8.41 ± 2.97	3-15
Occupational impact	13.66 ± 3.75	4-20
OIS total score	32.20 ± 8.63	10-50
Subjective sleep quality score	2.51 ± 0.67	1-4
Sleep latency score	1.55 ± 0.73	0-3
Sleep duration score	1.70 ± 0.46	0-2
Sleep efficiency score	0.02 ± 0.18	0-2
Sleep disturbances score	1.76 ± 0.53	1-3
Use of sleep medication score	1.03 ± 0.18	1-3
Daytime dysfunction score	1.91 ± 0.63	0-3
PSQI total score	10.33 ± 2.61	3-19

PSQI, Pittsburgh Sleep Quality Index; OIS, Occupational Identity Scale; SD, Standard Deviation.

### Effects of demographic variables on sleep quality in psychiatric night shift nurses

Significant differences in Pittsburgh Sleep Quality Index (PSQI) total scores were observed based on several night shift and lifestyle characteristics (**Table 1**). Participants with ≥6 night shifts per month (*p* = 0.021), an average of ≥3 night shifts per week (*p* = 0.005), an average night shift duration of >8 hours (*p* = 0.021), poor sleep quality the day before a night shift (*p* < 0.001), poor sleep quality the day after a night shift (p< 0.001), lack of daily exercise (*p* < 0.001), and irregular dietary patterns (*p* < 0.001) exhibited significantly poorer sleep quality compared to their counterparts.

### Association between sleep quality and occupational identity

The findings indicate that subjective sleep quality, sleep onset latency, sleep duration, sleep disturbance, and daytime dysfunction scores, together with the overall PSQI score, had significant negative correlations with the total OIS score and its dimensions (*p* < 0.05 or *p* < 0.001). This indicates that diminished sleep quality correlates with reduced levels of occupational identity. However, sleep efficiency and hypnotic medication scores were not significantly correlated with the total OIS score or its dimensions. (**Table 3**)

**Table 3:** Correlation coefficients between PSQI (total and components’) scores and OIS (total and dimensions’) scores.

Variable	OIS total score		Occupational cognition		Occupational evaluation		Occupational impact	
	r	p	r	p	r	p	r	p
Subjective sleep quality score	-0.343	<0.001	-0.322	<0.001	-0.322	<0.001	-0.306	<0.001
Sleep latency score	-0.252	<0.001	-0.238	<0.001	-0.23	<0.001	-0.299	<0.001
Sleep duration score	-0.241	<0.001	-0.218	<0.001	-0.214	<0.001	-0.230	<0.001
Sleep efficiency score	0.040	0.489	0.036	0.527	0.046	0.421	0.029	0.611
Sleep disturbances score	-0.205	<0.001	-0.224	<0.001	-0.153	<0.05	-0.193	<0.001
Use of sleep medication score	-0.038	0.508	-0.047	0.416	0.001	0.976	-0.056	0.330
Daytime dysfunction score	-0.279	<0.001	-0.279	<0.001	-0.241	<0.001	-0.253	<0.001
PSQI total score	-0.285	<0.001	-0.27	<0.001	-0.251	<0.001	-0.267	<0.001

PSQI, Pittsburgh Sleep Quality Index; OIS, Occupational Identity Scale.

### Factors influencing sleep quality

According to the multivariate logistic regression analysis (**Table 4**), sleep quality among psychiatric night-shift nurses is influenced by multiple factors. Working an average of 1 night shift per week (compared to ≥3 times per week) is a protective factor for sleep quality (OR = 0.431, 95% CI: 0.274-0.677, *p<* 0.001). Good sleep quality before (OR = 0.196, 95% CI: 0.056-0.690, *p* = 0.011) and after (OR = 0.409, 95% CI: 0.240–0.697, *p* = 0.001) night shifts significantly reduces the risk of poor sleep. Regular daily exercise (OR = 0.274, 95% CI: 0.127-0.591, *p* = 0.001) and regular diet (OR = 0.090, 95% CI: 0.018-0.450, *p* = 0.003) are also important protective factors. In addition, higher total scores on the occupational identity scale are associated with better sleep quality (OR = 0.948, 95% CI: 0.905-0.992, *p* = 0.021). The goodness-of-fit of the final logistic regression model was assessed using the Hosmer-Lemeshow test, which indicated no significant lack of fit (χ² = 8.21, *p* = 0.412).

**Table 4:** Logistic regression analysis of the multiple factors affecting sleep quality.

Variable	Group	B	SE	*P*	OR	95% CI for OR
Average number of night shifts per week	1	-0.842	0.230	<0.001	0.431	(0.274, 0.677)
	2	0.412	0.233	0.077	1.510	(0.956,2.386)
	≥3(reference)					
Sleep quality the day before a night shift	Good	-1.627	0.641	0.011	0.196	(0.056, 0.690)
	Average	-0.995	0.527	0.059	0.370	(0.132, 1.039)
	Poor(reference)					
Sleep quality the day after a night shift	Good	-0.894	0.272	0.001	0.409	(0.240, 0.697)
	Average	-0.324	0.218	0.137	0.723	(0.471, 1.110)
	Poor(reference)					
Daily exercise	Yes	-1.295	0.392	0.001	0.274	(0.127, 0.591)
	No(reference)					
Daily diet	Regular	-2.407	0.821	0.003	0.090	(0.018, 0.450)
	Irregular(reference)					
OIS total score		-0.054	0.023	0.021	0.948	(0.905, 0.992)

SE, Standard Error; OR, Odds Ratio; CI, Confidence Interval; OIS, Occupational Identity Scale.

## Discussion

### Generally poor sleep quality among psychiatric night shift nurses

The results showed that the prevalence of poor sleep quality among psychiatric nurses working night shifts was 81.06%. A previous cross-sectional study indicated that over 70% of psychiatric night shift nurses report poor sleep quality, which manifests as difficulty falling asleep, insufficient sleep duration, and daytime fatigue (23). In our study, elevated scores were particularly notable in the PSQI components of subjective sleep quality, sleep disturbance, and daytime dysfunction, which mirrors the symptomatic profile reported in the literature. Similar high rates of sleep problems have also been reported in surveys of nurses in general hospitals (24, 25). Critically, this widespread sleep impairment was significantly linked to specific work patterns and health behaviors within our cohort. Our univariate analysis revealed that nurses working higher-frequency night shifts and longer shifts reported significantly worse sleep quality. Importantly, the restorative capacity of sleep itself appears compromised, as both poor sleep quality before and after a night shift were strongly associated with higher PSQI scores. Beyond work schedules, modifiable lifestyle factors emerged as crucial correlates; irregular dietary patterns and a lack of regular daily exercise were both significantly associated with poorer sleep quality. The factors contributing to poor sleep quality in psychiatric night-shift nurses may be multifaceted. First, night shift work disrupts the endogenous circadian rhythm system and inhibits the normal secretion of melatonin, thereby affecting the initiation and maintenance of sleep (26). Secondly, psychiatric nurses are often exposed to patients’ neuropsychiatric symptoms, and such exposure may be intensified during night shifts, leading to increased psychological stress and enhanced stress responses, which in turn reduce deep sleep duration (23). Furthermore, some studies have suggested that anxiety and depressive symptoms caused by occupational characteristics may further disrupt sleep structure through neuroendocrine pathways (e.g., overactivation of the hypothalamic-pituitary-adrenal (HPA) axis) (27–29).

### Analysis of factors affecting sleep quality among psychiatric night shift nurses

Our multivariable analysis identified several significant independent predictors of poor sleep quality. These included sleep quality before and after night shifts, high weekly night shift frequency, irregular diet, lack of regular exercise, and lower occupational identity.

First, we found that nurses with poor sleep quality before and after night shifts faced an elevated risk of encountering sleep issues subsequent to working the night shift. Previous study has shown that sleep duration before night shifts significantly affects alertness and sleep quality after night shifts (30). Poor sleep quality before night shifts can disrupt the melatonin secretion cycle and activate the hypothalamic pituitary adrenal (HPA) axis, leading to impaired circadian rhythm regulation (31). Study has shown that night shift work often leads to circadian rhythm misalignment, characterized by a desynchronization between the endogenous melatonin rhythm and external work schedules. This circadian disruption hinders the body from reaching peak alertness during working hours, resulting in significant neurobehavioral performance deficits (32). Additionally, the delayed melatonin phase often conflicts with daytime recovery sleep, reducing sleep efficiency and prolonging sleep latency (33). Simultaneously, HPA axis activation induced by sleep deprivation elevates the baseline of cortisol secretion, further reducing the proportion of deep sleep (34). Even after catching up on eight hours of sleep after a night shift, nurses with poor sleep quality exhibited reduced sleep efficiency and a 40% increase in the incidence of daytime sleepiness (35). This suggests that the negative effects of poor sleep quality are cumulative.

Second, this study investigated the effects of the frequency of night shifts. It was found that high frequency night shifts were significantly associated with a high prevalence of poor sleep quality. This finding is consistent with a previous study, which has shown that the number of night shifts is positively associated with the severity of sleep disturbance (36). This may be closely related to circadian rhythm disruption, abnormal melatonin secretion, and the accumulation of long-term fatigue (35, 37). A 23-month follow-up cohort study showed that frequent night shifts can reduce nurses’ total daily sleep duration by approximately one hour (4). Furthermore, frequent night shifts are positively associated with the onset of depressive symptoms, further complicating sleep issues (34). These findings suggest that frequent night shifts may impair nurses’ sleep health through multiple physiological and psychological mechanisms.

Third, the daily diet of night-shift nurses affects their sleep quality. Due to high work stress and irregular meal times, psychiatric night-shift nurses are more prone to poor dietary quality, which may further exacerbate poor sleep quality (38). Lopez-Minguez et al. found that regular meal timing can improve metabolism and sleep by regulating circadian rhythm genes (39). Other studies indicate that irregular eating patterns correlate with poorer sleep quality (40, 41). Collectively, these findings suggest that dietary regularity may be positively related to sleep quality.

Fourth, night-shift nurses’ daily exercise habits also impact their sleep quality. Studies show that regular, moderate-intensity physical activity correlates with better sleep quality (42). Nurses who lack exercise are more prone to experiencing sleep disturbances (43). Exercise may positively impact sleep through multiple pathways, including regulating circadian rhythms, reducing stress levels, and decreasing nighttime awakenings (44). A review analysis shows that exercise can improve sleep quality by alleviating anxiety and depressive symptoms (45). However, the timing and intensity of exercise can also affect sleep differently. For example, high-intensity exercise close to bedtime may stimulate the sympathetic nervous system, potentially disrupting sleep (46).

### The relationship between sleep quality and occupational identity among psychiatric night shift nurses

Our study found that the total scores of the Pittsburgh Sleep Quality Index (PSQI) and its subscales were negatively correlated with the total score and subscales of the Occupational Identity Scale (OIS)(except for the sleep efficiency and use of sleep medication scores) (*P* < 0.001). Previous studies have shown that occupational identity is negatively correlated with the total sleep quality score. In other words, lower occupational identity is associated with poorer sleep quality (47). Nurses with poor sleep quality reported higher levels of occupational stress and anxiety (6). High occupational identity may alleviate stress by enhancing psychological resilience and positive coping strategies, thereby improving sleep quality (48). Nurses with a strong occupational identity are more likely to adopt proactive coping strategies and have significantly better sleep quality than those with a low identity (49). These findings suggest that occupational identity is not merely a psychological self-evaluation, but rather, it can translate into actual coping behaviors that positively influence sleep quality. Conversely, low occupational identity directly triggers emotional exhaustion and significantly increases the risk of sleep disorders (50, 51). This may be because nurses with low occupational identity lack the psychological support and coping resources necessary to face work-related stress, which makes them more prone to emotional distress and impaired sleep quality. Additionally, occupational identity improves sleep quality indirectly by promoting healthy behaviors, such as a regular diet and exercise, and by enhancing self-management awareness (52). These findings suggest that the impact of occupational identity on sleep quality extends beyond the psychological realm, positively influencing individual behavior and lifestyle. Furthermore, those possessing a robust occupational identity are better equipped to balance work and family conflicts, thereby reducing the risk of insomnia (53). Other studies have revealed a significant positive correlation between the sleep quality of night-shift nurses and depressive symptoms (34). Moreover, depressive states have been shown to further reduce occupational identity (54). Therefore, long-term sleep disorders can lead to negative evaluations of occupational value by weakening cognitive function and emotional regulation abilities and exacerbating occupational frustration (55–57).

### Clinical practice recommendations

Based on the findings of this study and existing evidence, we recommend a comprehensive management strategy to mitigate the risk of sleep disorders and enhance the occupational health of psychiatric night shift nurses. Hospital management should prioritize optimizing night shift schedules by limiting the number of consecutive night shifts, ensuring sufficient rest periods between shifts, and reducing high-frequency night shift arrangements to alleviate circadian rhythm disruption. Concurrently, it is essential to establish a routine sleep health monitoring and education system, such as regular screening using the PSQI and providing shift work specific sleep hygiene guidance. At the environmental level, comfortable dedicated rest areas should be made available for night-shift nurses, and evidence based psychological intervention programs (e.g., mindfulness training) should be implemented. Particularly important, given the protective role of occupational identity, active measures should be taken to strengthen nurses’ sense of professional value, including establishing mentorship programs, organizing case-sharing sessions, creating recognition mechanisms for night shift care, and conducting antistigma campaigns. Ultimately, fostering an organizational culture that values nurse well-being, offers clear career development pathways, and supports a positive team atmosphere can systematically promote better sleep quality and professional stability among nurses.

### Strengths and limitations

This study has several limitations. First, the sample size was limited to psychiatric nurses from a single region, which may restrict the generalizability of the findings. Second, the cross-sectional design precludes causal inference. Third, the diagnostic techniques for sleep problems utilized in this investigation predominantly depended on self-reported data, potentially introducing measurement bias. Subsequent study may use objective evaluation techniques, such as polysomnography, to complement the findings. Fourth, while we controlled for several key variables, the potential for unmeasured confounding (e.g., genetic predisposition to sleep disorders, detailed family responsibilities) cannot be ruled out. Future studies should aim to incorporate a broader range of potential confounding variables.

## Data Availability

The raw data supporting the conclusions of this article will be made available by the authors, without undue reservation.
